# Tabular context-aware optical character recognition and tabular data reconstruction for historical records

**DOI:** 10.1007/s10032-025-00543-9

**Published:** 2025-07-01

**Authors:** Loitongbam Gyanendro Singh, Stuart E. Middleton

**Affiliations:** School of Electronics and Computer Science, University of Southampton, Southampton, UK

**Keywords:** Optical Character Recognition, Tabular Structure Recognition, Semi-Supervised Learning, Historical Document Analysis, Data Annotation

## Abstract

Digitizing historical tabular records is essential for preserving and analyzing valuable data across various fields, but it presents challenges due to complex layouts, mixed text types, and degraded document quality. This paper introduces a comprehensive framework to address these issues through three key contributions. First, it presents UoS_Data_Rescue, a novel dataset of 1,113 historical logbooks with over 594,000 annotated text cells, designed to handle the complexities of handwritten entries, aging artifacts, and intricate layouts. Second, it proposes a novel context-aware text extraction approach (TrOCR-ctx) to reduce cascading errors during table digitization. Third, it proposes an enhanced end-to-end OCR pipeline that integrates TrOCR-ctx with ByT5, combining OCR and post-OCR correction in a unified training framework. This framework enables the system to produce both the raw OCR output and a corrected version in a single pass, improving recognition accuracy, particularly for multilingual and degraded text, within complex table digitization tasks. The model achieves superior performance with a 0.049 word error rate and a 0.035 character error rate, outperforming existing methods by up to 41% in OCR tasks and 10.74% in table reconstruction tasks. This framework offers a robust solution for large-scale digitization of tabular documents, extending its applications beyond climate records to other domains requiring structured document preservation. The dataset and implementation are available as open-source resources.

## Introduction

Digitizing historical tabular records, including climate data, agricultural logs, and financial ledgers, is essential for advancing research across various fields. These records contain valuable long-term data that help researchers identify historical patterns and trends. However, many records exist in analog formats, typically stored as tables in logbooks, ledgers, and archival documents. Extracting structured information from these sources poses unique challenges, especially for conventional Optical Character Recognition (OCR) systems, which are mainly designed for continuous text. These systems often struggle with the complex layouts of tables, leading to inaccuracies in capturing spatial relationships among cells, rows, and columns. This can result in fragmented or misaligned data, significantly reducing the quality and usability of the digitized information. Additionally, the scarcity of annotated historical logbook images further complicates the development of robust models for such tasks.

Recent advancements in transfer learning have shown substantial promise in addressing these challenges. Transfer learning allows models trained on large datasets to adapt to new, specific tasks with smaller datasets, thereby leveraging existing knowledge and features. Pre-trained models such as AlexNet [1] and Inception [2] have been successfully fine-tuned for OCR tasks in scenarios like script recognition and historical document digitization [3, 4]. Transformer-based models such as TrOCR [5] have also demonstrated effective text recognition capabilities for handwritten entries, making them particularly suitable for digitizing historical climate records. Similarly, deep learning models like DETR [6] and CascadeTabNet [7] have been applied for table structure recognition, enabling more accurate detection of cells, rows, and columns in complex tables [8–10]. These methods suggest that combining OCR advancements with structured data recognition can significantly improve the digitization of complex tabular data, even in resource-constrained environments.

Despite the technological advancements in the OCR model, building an end-to-end system for digitizing historical tabular logbooks remains expensive and resource-intensive, making it impractical for widespread use. While few studies have focused on smaller documents like receipts and business cards [11, 12], the challenge escalates when dealing with logbooks that contain over 1,000 densely packed cells. Transfer learning presents a potential solution by utilizing pre-trained models, yet current digitization pipelines are vulnerable to cascading errors. For instance, the Table Structure Recognition (TSR) model identifies and segments table regions in these pipelines, while the OCR model extracts text from these cells [8–10]. Failures in the TSR or OCR stage can propagate through the pipeline, compounding errors and reducing overall performance. Efforts to mitigate OCR errors often involve post-processing steps [13, 14]. Such a composite model, which integrates TrOCR with a language model such as ByT5 [15] for post-processing, showcases significant adaptability for handling historical documents that often contain degraded text perturbations.

In this paper, we address the challenges of digitizing historical tabular data through three key contributions. Firstly, we introduce UoS_Data_Rescue, a novel dataset comprising 1,113 historical logbooks with over 594,000 annotated text cells, specifically designed to capture the complexities of historical tabular data, including handwritten entries, aging artifacts, and intricate layouts. This dataset covers various text types (typed, mixed, handwritten), table layouts, and time periods (1860s to 1980s), providing a valuable resource for OCR and table structure recognition research. Secondly, we address cascading errors in the digitization process by proposing an enhanced training strategy for the TrOCR model pipeline, named TrOCR-ctx. This approach utilizes contextual information from neighboring cells to enhance text extraction. By doing so, TrOCR-ctx significantly reduces extraction errors and minimizes cascading failures, improving the accuracy of table reconstruction tasks. Finally, we incorporate ByT5 as an end-to-end model for post-OCR correction within the pipeline, enhancing the recognition of diverse languages, archaic terminology, and complex character sets. This setup significantly improves transcription accuracy across various table layouts, providing robust digitization for historical documents while effectively handling visual text perturbations [14].

By incorporating context awareness and addressing cascading errors through transfer learning, our model, TrOCR-ctx, consistently outperforms baseline OCR systems across diverse datasets, effectively handling complex table structures and mixed text formats (refer to Section 5). The key findings highlight the importance of incorporating neighboring cell information to reduce cascading errors and accurately capture spatial relationships within tables. While primarily focused on climate records, this methodology is adaptable to various fields requiring structured document digitization, such as financial archives, medical records, and historical census data. The research not only offers a practical framework for large-scale digitization of tabular documents but also enhances the accessibility of valuable historical records across diverse domains, identifying areas for future improvement in handling multi-cell layouts and multi-line text entries.

By sharing our code and the dataset^1^, we provide a practical framework for large-scaledigitization efforts, enhancing the accessibility of valuable historical records and offering tools forresearchers to advance data rescue initiatives across diverse fields.

The contributions of the paper are threefold:

i A novel dataset (UoS_Data_Rescue) containing 1,113 historical logbooks with over 594,000 annotated text cells, covering various text types, table layouts, and time periods from the 1860s to the 1980s, offering a valuable resource for OCR and table structure recognition research.
ii A novel fine-tuning approach (TrOCR-ctx) that utilizes contextual information from neighboring cells, significantly reducing cascading failures and thereby enhancing the accuracy of table reconstruction tasks.
iii We incorporate ByT5 as an end-to-end model for post-OCR correction within the pipeline, enhancing the recognition of diverse languages, archaic terminology, and complex character sets. This approach significantly improves transcription accuracy and robustness for historical document digitization.

## Related studies

The digitization of tabular documents from images has evolved significantly from traditional rule-based methods to advanced deep-learning models. Early approaches relied on predefined heuristics to identify tables based on visual layout features, effectively handling structured formats but struggling with irregular or complex layouts. As document diversity increased, the limitations of these rule-based systems became apparent, leading to adopting more adaptable machine-learning techniques. This review outlines the progression of techniques in this domain, highlighting key approaches and models that address the challenges of diverse document formats and the capabilities of OCR systems.

### Rule-based approaches

Optical Character Recognition (OCR) has been a foundational technology in digitizing tabular documents. Early approaches to table detection and extraction primarily relied on rule-based systems, utilizing predefined heuristics to identify tables based on visual layout features such as grid lines, alignment, and consistent spacing [16, 17]. These methods were effective for structured tables with regular formats, leveraging techniques like grid line detection, pattern recognition, and bounding box analysis in controlled scenarios. However, they often struggled with irregular or complex layouts and were inflexible when confronted with diverse or unstructured data.

While rule-based systems offer advantages in interpretability and precision for consistent formats, they are constrained by the complexity of rule creation and their inability to adapt to varying table structures. As the diversity of documents increased, the limitations of these systems became more pronounced, necessitating the adoption of more flexible machine learning (ML) techniques. These advanced approaches provide improved scalability and robustness for extracting tabular data from complex or unstructured documents, thereby enhancing the efficacy of OCR technologies in contemporary applications [18].

### Machine learning approaches

Machine learning techniques have significantly advanced table extraction, overcoming the limitations of traditional rule-based systems by offering greater adaptability and precision. By combining OCR with statistical models, these methods automate detection and recognition, enabling accurate whitespace identification and data extraction across diverse table types. Supervised learning approaches, such as Convolutional Neural Networks (CNNs) [19–21] and Support Vector Machines (SVMs) [22], have improved the identification of tables within complex layouts, with CNNs particularly adept at recognizing spatial structures in images.

The advent of deep learning has marked a significant leap forward in table extraction capabilities. End-to-end models like TableNet [23] and TC-OCR [9] integrate table detection and structure recognition into unified frameworks. TableNet treats these tasks as interdependent sub-problems within a single neural network, while TC-OCR combines state-of-the-art models such as DETR [6], CascadeTabNet [7, 8, 24], and PP-OCR v2 [25], effectively addressing variations in table styles and image distortions. Transformer-based models, including DeepDeSRT [26] and TableFormer [27], further enhance extraction capabilities. DeepDeSRT leverages a pre-trained ResNet-18 backbone to generate structured representations of tables, while TableFormer predicts bounding boxes for individual cells, facilitating precise content extraction from PDF documents. The integration of transfer learning allows these architectures to recognize both printed and handwritten text, making them particularly suitable for digitizing historical documents with diverse writing styles.

Transformer-based models have driven significant recent advances in OCR post-processing and end-to-end table extraction. Sequence-to-sequence models such as ByT5 and related transformer models have been effectively leveraged for robust post-OCR correction, substantially reducing character error rates in both modern and historical documents [14, 15]. The emergence of multimodal large language models, including Gemini 2.0 Flash^2^ and GPT-4o^3^, has further advanced the field by integrating visual and textual cues, setting new benchmarks for OCR correction in multilingual and noisy data scenarios [28].

Hybrid frameworks such as Table Transformer (TATR) [29, 30] and UniTable [31] provide unified architectures that jointly detect tables, recognize their structure, and extract cell content. In the domain of post-OCR correction, Chen et al. [13] combine TrOCR [5] with CharBERT [32], resulting in improved accuracy and reduced overcorrection, particularly for historical documents. Seth et al. [14] pair TrOCR with ByT5 [15], a byte-level transformer model, to address visual text perturbations. Rakshit et al. [33] present a comprehensive pipeline that integrates OCR (including TrOCR) with transformer-based NLP tools such as ByT5 and BART [34], refining outputs for printed and handwritten text.

Despite these advancements, challenges remain in handling diverse document formats, densely packed or nested cells, and noisy images, especially in historical documents with mixed handwriting and aging artifacts. Many state-of-the-art solutions require substantial computational resources and specific fine-tuning for different datasets, limiting their scalability and practical application. To address these challenges, our work introduces a specialized dataset UoS_Data_Rescue focused on historical climate logbook images and implements transfer learning strategies, particularly fine-tuning the TrOCR model to navigate the intricacies of historical records. By improving the context awareness of TrOCR (TrOCR-ctx) and integrating it with ByT5 for robust multilingual post-correction, our pipeline advances the field by enabling more resilient and accurate table extraction, particularly in resource-constrained and archival environments.

### Datasets for tabular data extraction

Several datasets have been developed to support research in Optical Character Recognition (OCR) and Tabular Data Extraction (TDE), each of which addresses different types of documents and challenges. TDE encompasses both the identification of table structures (such as cell boundaries and spatial relationships) and the extraction of cell content (text or numbers). Although some datasets focus exclusively on table structure recognition, a subtask of TDE, others provide both structure and content, which are crucial for end-to-end tabular data extraction.

In particular, datasets such as the ICDAR 2013 Table Competition [35], TableBank [36], and FinTabNet [37] are widely used benchmarks for the Table Structure Recognition (TSR) task. These datasets primarily provide annotations for table and cell boundaries, enabling models to learn how to segment and identify the structure of tables within documents. However, they typically do not include detailed transcriptions of cell content, and thus are focused on the structural aspect of tables rather than complete tabular data extraction.

In contrast, TDE datasets include both the structure of the table and information about cell content. PubTabNet, developed by IBM Research Australia, is an excellent example. It consists of scientific tables extracted from academic publications, annotated with HTML representations for ground-truth validation [38]. Although PubTabNet is valuable for OCR and table extraction tasks, it focuses primarily on structured, typed text, making it less suitable for historical documents that often contain handwritten entries, aging artifacts, and irregular layouts.

Other datasets such as CORD (Consolidated Receipt Dataset) [39] and SROIE (Scanned Receipt OCR and Information Extraction) [40] focus on receipt documents with relatively simple layouts and limited structural variability. CORD provides multilingual named entity annotations, while SROIE consists mostly of English-language receipts. These datasets are useful for evaluating OCR models in structured, modern documents, but do not address the complexities of historical tabular data, which often involve irregular layouts, handwritten text, and document degradation.

LayoutLM-based [20] datasets leverage multimodal learning by incorporating both textual content and spatial layout information, enabling models to better understand document structures. These datasets, often derived from existing OCR benchmarks, are primarily used for pre-training and fine-tuning LayoutLM models on tasks such as key information extraction, entity recognition, and document classification. They are particularly effective for modern documents with well-defined layouts, such as invoices, forms, and reports. However, they are not specifically designed for historical table extraction, as they lack variations in handwritten text, irregular table structures, and document degradation, which are common in archival records.

To address the gap in historical tabular datasets, we introduce UoS_Data_Rescue, a large-scale collection of 1,113 historical logbooks spanning diverse text types (typed, mixed, handwritten), intricate table structures, and aging artifacts throughout different periods (1860-1980s). Unlike modern datasets such as PubTabNet and LayoutLM-based datasets, which focus on structured, printed documents, or receipt-based datasets like CORD and SROIE, which contain relatively simple layouts, UoS_Data_Rescue explicitly captures the unique challenges of historical documents. The dataset features dense, compact tabular images with tightly packed handwritten and printed text, reflecting the formatting constraints of archival records. By preserving both table structures and diverse text content, this dataset enables a more rigorous evaluation of OCR models, particularly when handling handwritten text, degraded documents, and complex archival layouts.

## Research methodologies

**Fig. 1:**
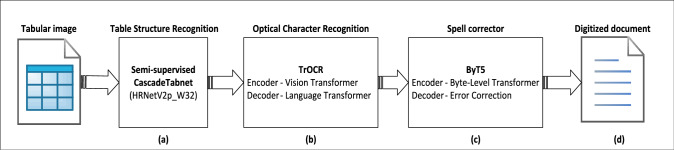
Block Diagram of the Tabular Data Extraction Pipeline. (a) Table and cell regions are detected using a semi-supervised Table Structure Recognition (TSR) model [7, 8]. (b) Each identified table cell and neighboring cell image is processed by TrOCR’s encoder-decoder architecture to generate OCR output text. (c) The OCR output is refined using ByT5, a byte-level transformer model, for post-OCR correction, ensuring accurate text extraction. (d) Finally, the tabular document is reconstructed based on spatial information from TSR and digitized text from OCR and spell correction, enabling precise tabular data representation

This section outlines the dataset and methodologies used to digitize historical tabular records, which are essential for preserving valuable data. These records often present challenges due to densely packed cells, handwritten entries, and complex layouts. To address these issues, we implement a systematic approach that integrates transfer learning for model fine-tuning and develops a robust tabular data reconstruction pipeline consisting of three components: (i) Table Structure Recognition (TSR), (ii) a customized tabular context-aware OCR model based on TrOCR, and (iii) a reconstruction module. Figure 1 presents the tabular data reconstruction pipeline. This integrated pipeline improves text extraction from noisy, aged records, enabling more effective digitization of tabular data.

**Table 1:** Overview of the UoS_Data_Rescue dataset, including the distribution of annotated and unlabeled logbook images across 34 regions. The checkmark ($^\checkmark$) indicates that the logbooks include a mix of handwritten and typed text, highlighting the diversity and complexity of the dataset

Location	Year	# Labelled images	Average cells/image	Average cells/image (hard-to-annotate)	# Unlabelled images
Sources: https://digital.nmla.metoffice.gov.uk/$^*$					
UK$^\checkmark$	1830-1930	97	208.959	3.804	–
Natal, Africa	1870	26	99.429	0.048	46
Artics$^\checkmark$	1880	82	477.122	30.220	–
Devon, UK$^\checkmark$	1890-1940	33	229.545	1.758	–
Ben Nevis, UK	1890	97	1511.247	11.557	–
UK and World$^\checkmark$	1900	93	622.793	31.141	1330
Philippines	1900	24	740.292	5.458	6077
India (NOAA)	1930	24	2197.429	7.476	380
India (MO)	1970	24	1971.667	17.208	276
Sources: https://digital.nmla.metoffice.gov.uk/					
Zanzibar$^\checkmark$	1881-1882	8	133.500	9.250	12
Blantyre$^\checkmark$	1882	–	–	–	6
Egypt$^\checkmark$	1885-1886	6	699.500	17.667	9
Morocco$^\checkmark$	1891	–	–	–	2
Sources: https://libguides.library.noaa.gov/weather-climate/foreign-climate					
Mauritius	1862-1972	34	227.559	0.235	13887
Algeria$^\checkmark$	1877-1968	113	90.947	7.292	22356
Madagascar	1889-1968	40	148.775	2.925	10035
Egypt	1900-1966	51	148.137	6.412	44199
Tunisia$^\checkmark$	1907-1932	29	119.207	4.034	2531
Uganda	1909-1937	5	188.000	0.000	456
Mozambique	1909-1968	44	289.364	2.909	19547
South Africa$^\checkmark$	1920-1982	74	109.946	0.757	36502
Libya	1922-1931	3	221.000	12.000	501
Kenya	1936-1937	–	–	–	31
Angola	1937-1952	–	–	–	1840
Namibia	1941-1948	–	–	–	52
Djibouti	1950-1974	–	–	–	1695
Cameroon	1950-1975	–	–	–	1830
Morocco	1954-1978	58	223.914	9.724	10575
Guinea-Bissau	1957-1972	–	–	–	3331
Sources: https://catalog.archives.gov					
Bear	1940	12	792.583	1.917	21
Tennessee	1946	36	880.611	1.472	17
Sources: http://archives-climat.fr					
Ambanja Août-décembre$^\checkmark$	1904	5	559.200	95.800	–
Diego-Suarez$^\checkmark$	1949	47	640.447	30.255	1
Tromelin$^\checkmark$	1956	48	651.396	33.021	–
Total	–	1113	–	–	177545

$^*$Original images sourced with permission from UK Met Office (MO), US NOAA and weatheerrescue.org (University of Reading) for the https://glosat.org/ project.

### UoS_Data_Rescue dataset

The dataset used in this study, UoS_Data_Rescue, comprises 1,113 scanned historical climate logbook images, with over 594,000 human annotations for cell boundaries and transcribed text. This dataset is specifically designed to support OCR and table structure recognition tasks by capturing diverse text types (typed, mixed, handwritten) and intricate table layouts. Table 1 provides a detailed overview of the distribution of unlabeled and annotated logbook images classified by year, region, and source. The source documents originate from prestigious institutions such as the UK Met Office^4^, US NOAA^5^, US naval ship logs^6^, and Meteo-France^7^.

The dataset captures challenges inherent in historical documents, including handwritten entries, faded artifacts, and complex layouts. Of particular note, 635 tabular images contain a mix of handwritten and typed text, spanning logbooks from the 1830s to the 1980s (as shown in Table 1). This diversity ensures that the dataset reflects the complexities of historical records. To achieve broad coverage and representation of these complexities, we employed a maximum variance sampling strategy that maximized variance in document format, handwriting styles, and time periods. This approach resulted in extensive coverage across low-density regions globally and high-density coverage in specific regions like Africa, aligning with the needs of climate scientists focused on these areas.

To ensure high-quality annotations, we use a crowd-sourced approach via the Appen platform^8^. Six annotators proficient in English and familiar with Latin alphabetical characters were employed to annotate the dataset in 18 batches, each containing approximately 1,500 images with no more than 40 cells per image. After each batch, quality evaluations were conducted to ensure accuracy; incorrectly annotated batches were re-run following evaluation. To promote annotation consistency, we adopted a majority vote protocol: *“an annotation was considered correct if at least three annotators agreed on the transcribed text”*. This approach helped mitigate individual errors and provided a measure of consensus. Recognizing that crowd-sourced annotators may lack expertise in transcribing historical handwriting styles, annotators flagged difficult-to-annotate cells for correction by domain experts. This rigorous annotation process ensures high-quality data for developing reliable tabular data reconstruction models.

To streamline the annotation process and support efficient table structure recognition, we used a pre-trained semi-supervised Table Structure Recognition (TSR) model based on CascadeTabNet [8]. The TSR model automatically detected table layouts and segmented text into individual cells, allowing annotators to focus on transcribing manageable portions of each image (*k* = 40 cells). For images containing more than *k* cells, additional copies were created with no more than *k* cells per image to simplify annotation. Annotators manually corrected errors produced by the TSR model to ensure high-quality data. Combined with its geographic coverage across 34 regions worldwide, this robust approach makes UoS_Data_Rescue an invaluable resource for advancing OCR capabilities and supporting the digitization of historical climate records.

In addition to the labelled scanned images, UoS_Data_Rescue also includes a large unlabelled collection of 177,545 scanned images. This extensive unlabelled dataset provides a rich resource for unsupervised learning and semi-supervised training methods, which can be leveraged to further improve OCR models by learning from the diverse patterns and structures present in these images.

**Table 2:** Distribution of training and testing data for fine-tuning TSR and OCR models, highlighting the unique characteristics of each dataset

Dataset	Table structure recognition		Optical character recognition		Average cells per image in test set
	#Training Images	#Testing Images	#Train text lines	#Test text lines	
UoS_Data_Rescue	1113	112	497045	97150	867.41
SROIE	1426	273	33626	18704	68.51
CORD	800	100	19367	2355	23.55
PubTabNet	6000$^*$	15115	26000$^+$	606719	40.14
ICDAR15	–	–	4468	2077	–

* The original PubTabNet dataset was released with 510K training samples

* Randomly selected 26000 text lines from the 6000 training samples

#### Dataset characteristics for OCR model training

To comprehensively evaluate the robustness of OCR models, we used a wide range of datasets that represent various text formats and layouts. Notably, we employed the in-house curated dataset, UoS_Data_Rescue, which stands out due to its exceptionally dense tables, approximately 10 times denser than those found in other datasets considered for evaluation. This density poses a unique challenge and fills a critical gap in existing datasets. Furthermore, we used the ICDAR 2015 Scene Text Recognition dataset [41, 42], and tabular-structured datasets such as CORD [19, 39, 43], SROIE [12, 20, 40], and PubTabNet [38, 44]. The ICDAR 2015 dataset serves as a benchmark for scene text detection and recognition, featuring images with text embedded in natural environments. The CORD and SROIE datasets focus on receipt images, primarily in English, and were used to fine-tune and assess the model’s performance in recognizing and extracting text from diverse receipt layouts. The PubTabNet dataset, consisting of scientific tables, was incorporated to fine-tune and test the model’s ability to manage complex tabular structures. Table 2 provides an overview of the data distribution for training and testing sets used to fine-tune the OCR model. These datasets collectively provide a robust set of scenarios for fine-tuning and evaluating OCR and tabular data reconstruction (TDR) tasks, ensuring the models are thoroughly tested on various text formats and layouts.

### Table structure recognition

To improve the accuracy of table structure recognition in historical climate logbooks, we fine-tuned a pre-trained Table Structure Recognition (TSR) model based on CascadeTabNet [8] using the annotated UoS_Data_Rescue dataset. The CascadeTabNet model employs a Cascade Mask R-CNN architecture [45] with a High-Resolution Network (HRNetV2p_W32) backbone [46], which extracts multi-scale features from document images and refines table detection through multiple stages. These stages predict the presence of tables and the precise boundaries of individual cells, making the model particularly effective for handling complex layouts and noisy, degraded images. The hyperparameters used to train CascadeTabNet are detailed in Appendix Table 8.

Since the trained model generates a limited number of table cells (up to 2000, including both positive and negative predictions), we implemented a method to infer missing cells based on the horizontal and vertical alignment of detected positive cells. This approach ensures complete table reconstruction by generating candidate cells where gaps are identified and aligning them with existing cells. This method is crucial in preprocessing data before OCR, as it accurately identifies table regions and defines cell boundaries. This robust table structure recognition provides a solid foundation for the downstream OCR model to perform precise text extraction. Ultimately, this improves the accuracy and reliability of tabular data reconstruction, supporting the successful digitization of historical climate records.

### Tabular context-aware optical character recognition

Building on the robust table structure recognition provided by the fine-tuned TSR model, our methodology adapts the TrOCR model specifically for extracting tabular data. Originally designed for continuous text recognition, TrOCR employs a Transformer-based architecture, utilizing a Vision Transformer (ViT) encoder to process images into visual embeddings, along with an autoregressive text decoder that generates text from these embeddings. For a comprehensive understanding of TrOCR’s architecture, readers are encouraged to refer to the original paper by Li et al. [5].

In this study, we enhanced TrOCR by incorporating context-awareness of neighboring table cells to improve its accuracy in digitizing historical tabular documents. Typically, TrOCR is fine-tuned on individual table cells or text line images. However, this approach can struggle with densely packed cells or handwritten text that crosses cell boundaries, leading to misalignment even when the TSR model accurately detects table layouts. To address this, we introduced a fine-tuning strategy that includes information from neighboring cells during training. Specifically, two additional images were generated for each table cell: one including the neighboring cell to the right and another including the cell below. The texts from neighboring cells were separated by boundary identifiers token [SEP], enriching the training dataset with contextual information and improving the model’s ability to handle irregular layouts and merged cells. We refer to this context-aware fine-tuning of TrOCR as TrOCR-ctx. The hyperparameters used to train TrOCR are detailed in Appendix Table 9.

During digitization, each detected table cell is expanded into two configurations: one with the neighboring cell to the right and another with the one below. The common text before [SEP] is extracted as the final output for the target cell. This approach significantly reduces cascading errors caused by isolated text lines or ambiguous boundaries by leveraging contextual cues during text extraction. By incorporating neighboring cell information, TrOCR-ctx develops a more comprehensive understanding of adjacent cells, leading to improved accuracy in recognizing text from challenging tabular configurations commonly found in historical documents.

### Post-processing OCR using a ByT5 model

TrOCR, while effective for many OCR tasks, struggles with multilingual text in historical documents due to irregular fonts, inconsistent spacing, and image degradation [13, 14]. These challenges often lead to tokenization errors or misrecognition of characters, particularly in archaic languages and non-standard character sets typical of historical records. To address this, we integrate ByT5, a byte-level Transformer model known for handling perturbed text, into the pipeline for post-OCR correction [14]. ByT5 processes text at the byte level, bypassing traditional tokenization, which allows it to handle diverse languages, archaic terminology, and complex character sets more effectively.

**Fig. 2:**
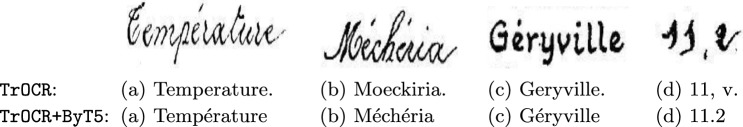
Examples of ByT5 post-OCR corrections on TrOCR outputs for historical text images, accurately recognizing complex characters in examples like ‘Température,’ ‘Méchéria,’ ‘Géryville,’ and numerical data ‘11.2.’

In our pipeline, the output of TrOCR (TrOCR-ctx) is fed into ByT5, which corrects recognition errors at the byte level. This enables ByT5 to refine text with non-standard characters and spelling variations, significantly improving transcription accuracy across various table layouts. For instance, as shown in Figure 2, ByT5 corrects TrOCR-ctx output by accurately transforming complex historical text such as “Température," “Méchéria," “Géryville," and “11.2" into their correct digital forms, handling archaic characters and diacritical marks with precision. This byte-level approach significantly enhances the accuracy of digitizing complex multilingual historical documents, making ByT5 particularly well-suited for challenging OCR tasks involving nuanced text recognition and correction.

### Tabular data reconstruction module

After extracting text from individual cells, the final step is reconstructing the digitized text to the original tabular format to preserve spatial and contextual relationships. Algorithm 1 outlines the reconstruction process. Specifically, the algorithm calculates the geometric centroids of each detected cell and organizes these centroids into horizontal and vertical lists corresponding to the rows and columns of the table. The recognized text is then assigned to the appropriate cell positions based on the proximity of the centroid, ensuring an accurate placement of the content within the reconstructed table.

**Algorithm 1:**
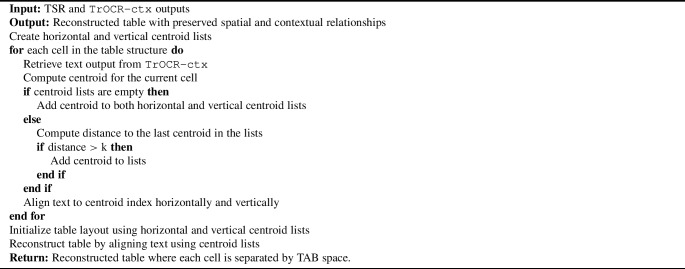
Tabular Data Reconstruction

Aligning table headers, particularly multicolumn headers, poses additional challenges for the centroid-based approach, which is otherwise effective for table bodies. To address this, we measure the deviation between each detected header cell centroid and its expected position within the table body grid, aligning each header to the nearest table body centroid. This metric helps identify and correct alignment errors in complex header scenarios, improving the overall accuracy of the reconstruction. This step is essential to maintain the integrity of the digitized data. The reconstruction module combines the outputs of the TSR and fine-tuned TrOCR-ctx models to accurately recreate the table layout, ensuring alignment with the original structure. This alignment improves the usability and accuracy of the digitized data, making them more valuable for research and analysis. Ultimately, the module enhances the fidelity of the digitization process, preserving historical data in its proper form.

## Experimental setup

Existing end-to-end models [11, 12] often require substantial computational resources, which limits their practical use on large, densely structured historical datasets such as UoS_Data_Rescue. Due to these constraints, we were unable to perform direct comparisons with these state-of-the-art end-to-end methods in this study. Instead, our modular pipeline is designed to be more resource-efficient and adaptable to environments with limited computational capacity. To thoroughly evaluate the robustness and effectiveness of our tabular data reconstruction pipeline, we compare the performance of the TrOCR-ctx model with three other fine-tuned OCR models: TrOCR [5], ABINet [41], and PP-OCRv2 (PaddleOCR) [25]. By benchmarking across a diverse set of datasets, our evaluation provides a comprehensive assessment of each model’s ability to generalize to different document types while highlighting the practicality and adaptability of our approach for real-world historical document digitization.

### Evaluation metrics

To comprehensively evaluate the performance of the OCR models and the overall TDR pipeline, we used a variety of evaluation metrics tailored to both the TSR and OCR tasks. These metrics offer insights into the accuracy, precision, and robustness of the models in various aspects of table structure detection, text extraction, and reconstruction.

#### Evaluation metrics for table structure recognition

For TSR, the evaluation focuses on how accurately the model detects table structures, including cell boundaries and overall layout. In this study, we use the Weighted Average F1 (wF1) score as the primary metric [47]:

1 $$\begin{aligned} \text {wF1} = \sum _{i} w_i \cdot \frac{2 \cdot \text {Precision}_i \cdot \text {Recall}_i}{\text {Precision}_i + \text {Recall}_i} \end{aligned}$$

Here, $w_i$ represents the weight for each Intersection over Union (IoU) threshold *i*, and ${Precision}_i$ and ${Recall}_i$ are the precision and recall at the $i^{th}$ IoU threshold. The IoU thresholds are set to 0.6, 0.7, 0.8, and 0.9. A prediction is considered correct if it meets or exceeds these thresholds, ensuring that precision and recall are balanced across varying levels of overlap between predicted and actual table structures.

#### Evaluation metrics for optical character recognition

To evaluate the robustness of the OCR models, we used a diverse set of metrics tailored to assess different aspects of performance, particularly in handling tabular structured documents. These metrics include ROUGE-L [48], Word Error Rate (WER) [49], Character Error Rate (CER) [49], Exact Match (EM) [50], and F1-scores at both character and token levels [50]. Each metric was selected for its ability to provide unique insights into OCR model performance. ROUGE-L measures sequence-level accuracy by comparing the longest common subsequence between predicted text and ground truth, making it useful for evaluating longer text sequences. WER and CER are standard OCR evaluation metrics that quantify word and character-level errors, respectively, offering a granular view of text recognition accuracy. Exact Match (EM) provides a strict evaluation by checking if the predicted text exactly matches the ground truth, which is critical for assessing perfect OCR output. Finally, F1-scores at both character and token levels balance precision and recall, capturing partial matches where minor errors occur. The F1-score is calculated as follows:

$$\begin{aligned} \text {F1-score} = 2 \times \frac{\text {Precision} \times \text {Recall}}{\text {Precision} + \text {Recall}} \end{aligned}$$

where $\text {Precision} = \frac{\texttt {M}}{\texttt {P}}$ and $\text {Recall} = \frac{\texttt {M}}{\texttt {G}}$. This formula applies to both evaluation levels. For the Character-Level F1-Score, M, P, and G represent matched, predicted, and ground truth characters, respectively. For the Token-Level F1-Score, these variables denote matched, predicted, and ground truth tokens. These metrics comprehensively evaluate the OCR model’s robustness in handling complex historical tabular data.

#### Overall Performance Metrics

By integrating evaluation metrics for both TSR and OCR, we obtain a comprehensive assessment of the entire tabular data reconstruction pipeline. The weighted F1-score allows us to evaluate the accuracy of table structure detection, including cell boundaries and overall layout. Meanwhile, OCR metrics like Rouge-L, Word Error Rate (WER), Character Error Rate (CER), and F1-scores at both character and token levels provide detailed insights into text extraction accuracy. These metrics capture exact matches and account for partial correctness, which is crucial for handling complex historical documents often plagued by noisy or degraded data. This combination ensures a holistic evaluation of the digitization process, reflecting the high-level performance of table detection and fine-grained text recognition accuracy. Ultimately, this multifaceted evaluation approach allows us to pinpoint specific areas for improvement while preserving the integrity of tabular documents.

**Fig. 3:**
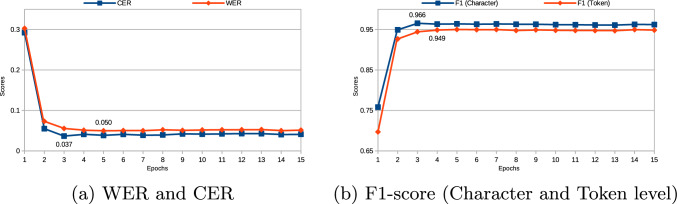
Performance evaluation of TrOCR-ctx per epoch, showing the progression of WER, CER, and F1-scores at both character and token levels. The labeled epoch indicates the peak performance among the 15 epochs, with the model converging after approximately 3 epochs

### Experiment runtime and hardware specifications

The experiments were conducted on a multi-GPU system with two NVIDIA A100 GPUs, each with 80 GB of memory. This setup was essential to handle the extensive datasets and complex computations required for training TrOCR-ctx (refer to Table 2). Training TrOCR-ctx for 15 epochs took approximately two weeks, reflecting the computational demands of incorporating context-aware text extraction. In comparison, the baseline TrOCR model completed 15 epochs in about one week, demonstrating that introducing additional context-aware samples in TrOCR-ctx requires more processing time but improves performance across various datasets. Figure 3 illustrates the performance of TrOCR-ctx per epoch, showing improvements in word error rate (WER), character error rate (CER), and F1-scores at both character and token levels. Notably, the model converges approximately after the third epoch, indicating efficient learning despite the computational intensity.

**Table 3:** Performance comparison of TrOCR-ctx, TrOCR, Abinet, and PP-OCRv2 models on UoS_Data_Rescue, CORD, SROIE, PubTabNet, and ICDAR 2015 datasets using segmented text lines. Evaluation metrics include Rouge-L, Word Error Rate (WER), Character Error Rate (CER), Exact Match, and F1-scores at both character and token levels

OCR Model	Rouge-L	WER	CER	EM	F1-score (Char)	F1-score (Token)
	UoS_Data_Rescue					
TrOCR	0.849	0.055	0.047	0.825	0.963	0.945
TrOCR-ctx	**0.857**	**0.049**	**0.035**	**0.847**	**0.966**	**0.951**
Abinet	0.545	0.557	0.346	0.432	0.681	0.449
PP-OCRv2	0.812	0.348	0.178	0.646	0.825	0.666
	CORD					
TrOCR	0.898	0.168	0.056	0.802	0.946	0.834
TrOCR-ctx	**0.957**	**0.034**	**0.016**	**0.833**	**0.985**	**0.986**
Abinet	0.520	0.356	0.304	0.574	0.710	0.644
PP-OCRv2	0.789	0.114	0.144	0.746	0.897	0.886
	SROIE					
TrOCR	0.919	0.053	0.044	0.830	0.984	0.947
TrOCR-ctx	**0.940**	**0.033**	**0.014**	**0.849**	**0.988**	**0.967**
Abinet	0.872	0.629	0.497	0.301	0.507	0.381
PP-OCRv2	0.882	0.432	0.235	0.493	0.777	0.577
	PubTabNet					
TrOCR	0.878	0.141	0.069	0.748	0.940	0.859
TrOCR-ctx	**0.913**	**0.091**	**0.067**	**0.789**	**0.965**	**0.909**
Abinet	0.315	0.813	0.450	0.153	0.598	0.197
PP-OCRv2	0.833	0.131	0.097	0.700	0.915	0.877
	ICDAR 2015					
TrOCR	**0.777**	**0.245**	0.102	0.744	0.904	0.750
TrOCR-ctx	0.776	0.250	0.102	**0.749**	**0.905**	**0.755**
Abinet	0.151	0.741	0.624	0.259	0.436	0.259
PP-OCRv2	0.665	0.374	0.178	0.626	0.831	0.627

**Table 4:** Performance evaluation of TrOCR-ctx model on Tabular Data Reconstruction across UoS_Data_Rescue, CORD, SROIE, and PubTabNet datasets. Precision and Recall for table structure recognition are calculated based on an IoU threshold $\ge$ 0.6

	Table structure recognition			Tabular data reconstruction						
Dataset	P	R	wF1	Rouge-L	WER	CER	EM	F1 (Char)		F1 (Token)
Without contextual information contextual information (TrOCR)										
UoS_Data_Rescue	0.742	0.919	0.805	0.771	0.281	0.254	0.719	0.819		0.719
CORD	0.970	0.715	0.798	0.890	0.043	0.031	0.863	0.890		0.863
SROIE	0.805	0.796	0.785	0.847	0.046	0.039	0.819	0.869		0.819
PubTabNet	0.959	0.814	0.869	0.618	0.584	0.593	0.408	0.525		0.408
With contextual information contextual information (TrOCR-ctx without ByT5 model)										
UoS_Data_Rescue	0.742	0.919	0.805	0.778	0.258	0.232	0.742	0.824 ($\Delta 0.61\%$)		0.742 ($\Delta 3.20\%$)
CORD	0.970	0.715	0.798	0.917	0.035	0.023	0.895	0.913 ($\Delta 2.58\%$)		0.895 ($\Delta 3.71\%$)
SROIE	0.805	0.796	0.785	0.872	0.025	0.023	0.875	0.909 ($\Delta 4.60\%$)		0.875 ($\Delta 6.84\%$)
PubTabNet	0.959	0.814	0.869	0.636	0.584	0.593	0.416	0.527 ($\Delta 0.38\%$)		0.416 ($\Delta 1.96\%$)
With contextual information contextual information (TrOCR-ctx with ByT5 model)										
UoS_Data_Rescue	0.742	0.919	0.805	0.809	0.245	0.213	0.755	0.850 ($\Delta 3.79\%$)		0.755 ($\Delta 5.01\%$)
CORD	0.970	0.715	0.798	0.917	0.023	0.025	0.914	0.921 ($\Delta 3.48\%$)		0.914 ($\Delta 5.91\%$)
SROIE	0.805	0.796	0.785	0.908	0.023	0.022	0.907	0.914 ($\Delta 5.18\%$)		0.907 ($\Delta 10.74\%$)
PubTabNet	0.959	0.814	0.869	0.640	0.592	0.594	0.426	0.536 ($\Delta 2.10\%$)		0.426 ($\Delta 4.41\%$)

## Results and discussion

### OCR performance analysis

Table 3 presents the evaluation of OCR models—TrOCR-ctx, TrOCR, Abinet, and PP-OCRv2—across diverse datasets including UoS_Data_Rescue, ICDAR 2015, CORD, SROIE, and PubTabNet. These datasets were used to assess the robustness and accuracy in handling various text formats and layouts, with the evaluation conducted on properly segmented text lines from the test sets (refer Table 2). TrOCR-ctx, fine-tuned with context-aware patches, consistently achieved the highest performance across all datasets. Its superior F1-scores, ranging from 0.755 to 0.986, highlight its ability to accurately recognize text in challenging scenarios, such as mixed handwritten and typed text or complex table structures. This model excelled in handling historical tabular data, achieving F1-scores of 0.951 for UoS_Data_Rescue, 0.986 for CORD, 0.967 for SROIE, 0.909 for PubTabNet, and 0.755 for ICDAR 2015. Additionally, it demonstrated low CER, ranging from 0.014 (SROIE) to 0.102 (ICDAR 2015), and high Rouge-L scores from 0.776 (ICDAR 2015) to 0.957 (CORD). Among the other OCR models, TrOCR performed better than Abinet and PP-OCRv2 but lagged behind TrOCR-ctx. TrOCR achieved F1-scores of 0.945 on UoS_Data_Rescue, 0.834 on CORD, 0.947 on SROIE, 0.859 on PubTabNet, and 0.750 on ICDAR 2015. Its CER ranged from 0.044 to 0.102, and its Rouge-L scores from 0.777 to 0.919, indicating solid performance but with room for improvement compared to TrOCR-ctx. The lower performance of Abinet and PP-OCRv2 on datasets with complex historical data emphasizes context awareness, underscoring the importance of robust models like TrOCR-ctx for such tasks.

### Tabular data reconstruction performance

Following the superior OCR performance of TrOCR-ctx, we conducted a detailed evaluation of its tabular data reconstruction capabilities in multiple datasets, including UoS_Data_Rescue, CORD, SROIE, and PubTabNet. The results presented in Table 4, demonstrate a clear performance advantage of TrOCR-ctx over the baseline TrOCR model, primarily due to its context-aware fine-tuning. By incorporating contextual information during training, TrOCR-ctx consistently outperformed the baseline in all data sets. First, we evaluated the performance of TrOCR-ctx without post-correction from ByT5. The inclusion of contextual information in text extraction significantly improved performance compared to the non-contextual TrOCR model. Specifically, TrOCR-ctx achieved 0.61% and 3.20% improvement in the F1 scores at the character level and token level, respectively, on UoS_Data_Rescue. Similarly, it outperformed TrOCR in CORD by 2.58% and 3.71%, in SROIE by 4.60% and 6.84%, and in PubTabNet by 0.38% and 1.96%, at the character level and the token level, respectively. Next, we evaluated the impact of adding a post-OCR correction using ByT5 to further refine the output of TrOCR-ctx. This additional step resulted in significant performance improvements in all datasets. Specifically, with ByT5 post-correction applied, TrOCR-ctx achieved a 3.79% and 5.01% improvement in F1-scores at the character-level and token-level on UoS_Data_Rescue, respectively. Similarly, it outperformed TrOCR in CORD by 3.48% and 5.91%, in SROIE by 5.18% and 10.74%, and in PubTabNet by 2.1% and 4.41%, at the character-level and the token-level. These results highlight the effectiveness of context-sensitive fine-tuning in TrOCR-ctx to improve OCR accuracy and demonstrate the additional benefits of integrating ByT5 for post-OCR correction in handling complex tabular data extraction tasks.

However, both OCR performances on the PubTabNet dataset were notably lower than others, despite having good performance on properly segmented text lines. This lower performance can be attributed to two main factors. First, the Table Structure Recognition (TSR) model struggled with accurately aligning table layouts, even when the Intersection over Union (IoU) score exceeded 0.6. This misalignment significantly impacted the overall performance of the OCR system. For context, we used a randomly selected subset of 6,000 images for training and evaluated the model on a test set of 15,115 images from PubTabNet. Second, TrOCR faced difficulties processing longer multi-line text entries, which were abundant in PubTabNet. Similar issues were observed in some logbooks within the UoS_Data_Rescue dataset containing multi-line text entries. Additional challenges included handling complex table structures and irregular cell boundaries, which further affected performance on both datasets. These findings highlight the need to improve table layout alignment and multi-line text recognition to enhance OCR accuracy for complex tabular data reconstruction tasks.

**Table 5:** Performance breakdown of TrOCR-ctx on the UoS_Data_Rescue dataset, evaluating various aspects such as full tables, table body, individual text-only cells, and number-only cells. The table body, text-only, and number-only cells are further categorized based on a mix of handwritten (Mixed) and typed (Typed) text

	Table structure recognition			Tabular data reconstruction					
Dataset	P	R	F1	Rouge-L	WER	CER	EM	F1 (Char)	F1 (Token)
Full table	0.742	0.919	0.805	0.809	0.245	0.213	0.755	0.850	0.755
Table body (Mixed)	0.783	0.878	0.820	0.770	0.207	0.199	0.793	0.867	0.793
Table body (Typed)	0.827	0.964	0.885	0.897	0.108	0.121	0.892	0.937	0.892
Text (Mixed)	0.646	0.860	0.707	0.782	0.284	0.287	0.716	0.817	0.716
Text (Typed)	0.659	0.811	0.695	0.772	0.249	0.256	0.751	0.836	0.751
Number (Mixed)	0.774	0.863	0.805	0.943	0.076	0.056	0.924	0.954	0.924
Number (Typed)	0.820	0.920	0.858	0.969	0.047	0.039	0.953	0.974	0.953

To gain deeper insights into TrOCR-ctx’s performance, we conducted a detailed evaluation by combining TrOCR’s context-aware OCR capabilities with the post-OCR correction provided by the ByT5 model. The analysis focused on various table regions within historical logbook images from the UoS_Data_Rescue dataset. Table 5 presents a performance breakdown, highlighting the model’s ability to handle dense tables and complex data, particularly those containing mixed handwritten content. In the full table evaluation, including the header and body, TrOCR-ctx achieved F1-scores of 0.850 at the character level and 0.755 at the token level. This demonstrates the model’s ability to capture the overall table structure while maintaining content accuracy across the image. However, when focusing solely on the table body—where most of the critical information in historical logbooks resides—the model’s performance improved, suggesting that alignment issues with header cells may impact overall reconstruction accuracy. For table bodies containing a mix of handwritten and typed text, TrOCR-ctx achieved an F1-score of 0.793 at the token level. When evaluating only typed text table bodies, the model reached an impressive F1-score of 0.892 at the token level, indicating its superior handling of typed content compared to handwritten-mixed entries.

Analysis of different types of cell content extraction performance (text vs. numbers) revealed significant disparities between text and numerical content processing. When evaluating different types of cell content, the model showed exceptional performance on numbered cells, achieving an F1-score of 0.924 for handwritten-mixed content and 0.953 for typed-only content at the token level. In contrast, text cells scored lower, with F1-scores of 0.719 for handwritten-mixed content and 0.751 for typed-only content at the token level. While this variation suggests that handwritten text and complex layouts in text cells present more difficulty, the model’s strong performance on numbered cells demonstrates its potential. With further fine-tuning and targeted improvements, particularly in handling handwritten text, TrOCR-ctx can continue to advance in precision and robustness for the digitization of historical documents.

**Table 6:** Performance of TrOCR-ctx across different regions in the UoS_Data_Rescue dataset, providing a logbook-wise analysis to evaluate how the model performs on various logbook types and regions

	Table structure recognition			Tabular data reconstruction					
Regions	P	R	F1	Rouge-L	WER	CER	EM	F1 (Char)	F1 (Token)
Tromelin$^\checkmark$	0.903	0.903	0.897	0.928	0.088	0.081	0.912	0.949	0.912
Diego-Suarez$^\checkmark$	0.836	0.866	0.840	0.902	0.115	0.109	0.885	0.936	0.885
UK$^\checkmark$	0.666	0.998	0.782	0.939	0.131	0.121	0.869	0.942	0.869
Ambanja Août-décembre$^\checkmark$	0.588	0.944	0.719	0.922	0.131	0.068	0.869	0.945	0.869
South Africa$^\checkmark$	0.640	0.954	0.764	0.909	0.133	0.178	0.867	0.895	0.867
Natal, Africa	0.757	0.697	0.720	0.951	0.135	0.117	0.865	0.931	0.865
Tunisia$^\checkmark$	0.853	0.935	0.890	0.906	0.153	0.215	0.847	0.904	0.847
Zanzibar$^\checkmark$	0.912	0.946	0.928	0.905	0.156	0.162	0.844	0.884	0.844
Algeria$^\checkmark$	0.787	0.904	0.832	0.913	0.175	0.135	0.825	0.905	0.825
Tennessee	0.414	0.914	0.530	0.822	0.188	0.218	0.812	0.860	0.812
Libya	0.782	0.881	0.819	0.898	0.199	0.205	0.801	0.858	0.801
Bear	0.352	0.875	0.488	0.834	0.201	0.177	0.799	0.869	0.799
Devon, UK	0.907	0.986	0.943	0.839	0.202	0.182	0.798	0.861	0.798
Arctic$^\checkmark$	0.737	0.969	0.834	0.663	0.313	0.327	0.687	0.727	0.687
Egypt$^*$ $^\checkmark$	0.420	0.896	0.552	0.791	0.339	0.260	0.661	0.800	0.661
India$^*$	0.781	0.915	0.841	0.663	0.345	0.297	0.655	0.789	0.655
Uganda	0.838	0.893	0.864	0.856	0.362	0.460	0.638	0.693	0.638
Morocco	0.508	0.722	0.589	0.706	0.377	0.481	0.623	0.710	0.623
Egypt$^+$	0.779	0.822	0.783	0.854	0.423	0.381	0.577	0.744	0.577
Madagascar	0.819	0.923	0.864	0.778	0.426	0.598	0.574	0.627	0.574
Mozambique	0.729	0.765	0.730	0.745	0.469	0.770	0.531	0.603	0.531
India$^+$	0.884	0.876	0.879	0.566	0.473	0.576	0.527	0.665	0.527
UK and World$^\checkmark$	0.821	0.863	0.834	0.547	0.489	0.812	0.511	0.633	0.511
Mauritius	0.846	0.831	0.834	0.786	0.506	0.725	0.494	0.583	0.494
Ben Nevis, UK	0.981	0.845	0.907	0.627	0.520	0.510	0.480	0.668	0.480
Philippines	0.869	0.858	0.842	0.406	0.711	0.652	0.289	0.482	0.289

$^*$ The source of these logbooks is from the UK Met Office

$^+$ The source of these logbooks are from the US NOAA

$^\checkmark$ The logbooks contain a mix of handwritten and typed text

To gain deeper insights into regional variations in logbook layouts, we conducted a logbook-wise evaluation of TrOCR-ctx performance across different sources and layouts. Table 6 provides a detailed breakdown of performance, sorted by F1-scores at the token level, revealing significant differences between logbooks. Logbooks with simpler layouts and clearer handwriting consistently achieved higher F1-scores, while those with more complex layouts or degraded handwriting presented greater challenges for the model. Factors contributing to these variations include densely packed table cells (e.g., India$^+$, Ben Nevis), irregular layout complexity (e.g., UK and World, Mozambique), mixed content with varying handwriting quality (e.g., UK and World), and dense multi-line text entries (e.g., Philippines). These factors made it more difficult for the model to accurately reconstruct tables in certain cases.

**Fig. 4:**
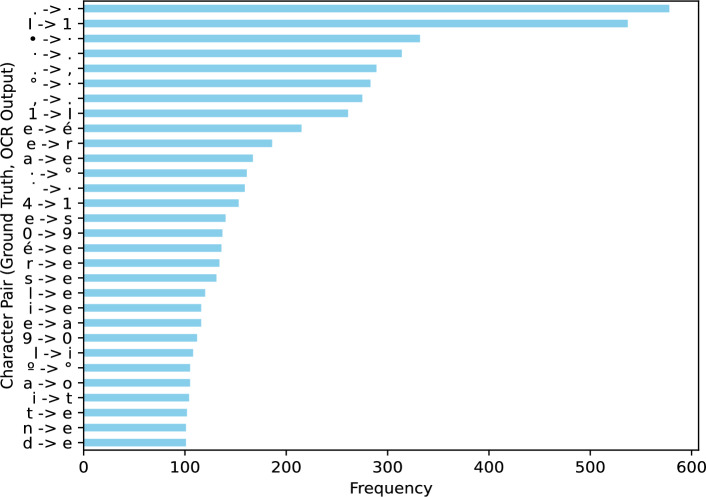
Bar chart illustrating the frequency of TrOCR-ctx substitution errors, showcasing common character misrecognition between the ground truth and the OCR-predicted output. This breakdown highlights the top 30 frequently substituted character pairs, providing insights into recurring OCR inaccuracies and potential areas for model improvement

To better understand these performance variations, we conducted a detailed error analysis of the OCR output. This analysis revealed several key challenges. First, a notable issue arose from the crowdsourced annotation process, particularly in the representation of numerical data common in climate logbooks. For example, annotators frequently misinterpreted decimal points or periods (.) as interpuncts (.) and periods as degree symbols ($\circ$) due to historical handwriting styles. While these inconsistencies reflect the authentic appearance of historical records, they introduced additional complexity in evaluating the model’s performance on numerical data. Second, we performed an error analysis focusing on character-level substitution errors. Figure 4 illustrates frequent character substitutions encountered during digitizing the UoS_Data_Rescue dataset. These substitutions offer valuable insights into common misrecognition patterns. High-frequency errors, such as (., .), ($\circ$, .), and (I, 1), indicate challenges in distinguishing visually similar characters, particularly those with fine distinctions in handwritten dots, strokes, and numerals. Additionally, substitutions like (4, 1) and (0, 9) suggest difficulties recognizing certain numeric characters, likely due to overlapping or similarly shaped glyphs in cursive or non-standard handwriting styles. Character pairs like (e, r) and (e, é) further highlight issues with recognizing subtle handwriting variations, diacritical marks, and capitalization—common challenges in historical texts with irregular handwriting and faded ink.

These patterns emphasize the need to further enhance the TrOCR-ctx fine-tuning to improve accuracy in recognizing frequently misinterpreted characters in handwritten documents. Understanding these variations and annotation challenges will guide future improvements in both OCR and TSR models, particularly in addressing the unique challenges posed by historical documents with intricate layouts, poor handwriting quality, and specialized numerical notation.

### Discussion

Performance analysis provides several key insights into the performance of TrOCR-ctx for tabular data reconstruction. One notable finding is the alignment issue between full tables and table bodies, where discrepancies in header alignment negatively impact digitization accuracy. While TrOCR-ctx performs well on properly segmented text lines (refer to Table 3), it struggles to maintain spatial relationships when headers are involved, leading to misaligned data during reconstruction. Incorporating contextual information from surrounding cells significantly enhances the model’s ability to capture spatial relationships, particularly in historical documents with intricate layouts.

The error analysis, as illustrated in Figure 4, reveals common character substitution errors made by TrOCR-ctx. High-frequency errors, such as confusing visually similar characters (e.g., ‘.’ and ‘.’, ‘I’ and ’1’), indicate difficulty distinguishing fine details in handwritten text. Numeric character recognition also presents difficulties, with substitutions like ‘4’ for ‘1’ and ‘0’ for ‘9’ suggesting issues with overlapping or similarly shaped glyphs in cursive or non-standard handwriting styles.

Performance varies significantly between numbered and text cells. Numbered cells consistently achieved higher F1-scores than text cells, especially when dealing with handwritten entries (refer to Table 5). For instance, numbered cells achieved F1-scores of 0.924 for handwritten-mixed content and 0.953 for typed-only content, compared to 0.719 and 0.751 for text cells, respectively. This disparity highlights the ongoing challenges in recognizing complex handwritten text and layouts. Additionally, TrOCR-ctx performed better on typed text than handwritten-mixed entries, which often cross cell boundaries and complicate alignment. This emphasizes the importance of refining Table Structure Recognition (TSR) to better handle handwritten content. Lastly, the logbook-wise analysis (refer to Table 6) revealed performance variations based on layout complexity and handwriting quality, offering further opportunities for improvement.

In summary, while TrOCR-ctx demonstrates significant advancements in handling complex tabular data through context-aware fine-tuning, challenges related to alignment, handwritten text recognition, long multi-line text entries, and character-level distinctions still need to be addressed for further optimization. The error analysis provides valuable insights for future improvements, particularly in enhancing the model’s ability to distinguish visually similar characters and handle the intricacies of handwritten text in historical documents.

## Conclusion and future work

This study on the digitization of historical tabular records using the context-aware TrOCR model, particularly TrOCR-ctx, has demonstrated promising results. By introducing the specialized UoS_Data_Rescue historical climate logbook dataset, we provided a robust foundation for training and evaluating OCR models tailored to the complexities of historical tabular data. Through comprehensive evaluations across multiple datasets, TrOCR-ctx consistently outperformed baseline models, proving its effectiveness in recognizing text within complex table structures and diverse formats, including mixed handwritten and typed entries. Key findings highlight the importance of context-awareness in OCR and table reconstruction. By incorporating information from neighboring cells, TrOCR-ctx reduced recognition errors and relationships more accurately within the tables. However, challenges remain, particularly in aligning header cells and recognizing handwritten text that crosses cell boundaries. The model’s strong performance on typed text underscores its potential for digitizing historical records with well-formatted text while also pointing to areas for improvement in handling handwritten entries.

Moving forward, future work will focus on improving the alignment of table cells, particularly addressing issues with header cells and improving the recognition of handwritten text—areas where TrOCR-ctx still faces challenges. This could involve fine-tuning models on more diverse handwritten datasets and developing advanced preprocessing techniques to better handle complex layouts. Expanding the UoS_Data_Rescue dataset with more intricate layouts and varied text styles will provide a broader training ground for OCR models. Additionally, re-correcting the ground truth based on identified crowdsourced annotation errors, such as misinterpretations of numerical data (e.g., decimal points misread as interpuncts or degree symbols), could enhance the accuracy of future evaluations. Efforts will also be made to improve model robustness against noise and distortions, optimize scalability for large-scale digitization projects, and incorporate feedback from domain experts to further refine the model’s performance. Finally, rigorous cross-validation will ensure the model’s generalization across diverse datasets and real-world scenarios, ensuring continued advancements in historical document digitization.

## Figures and Tables

**Fig. 5:**
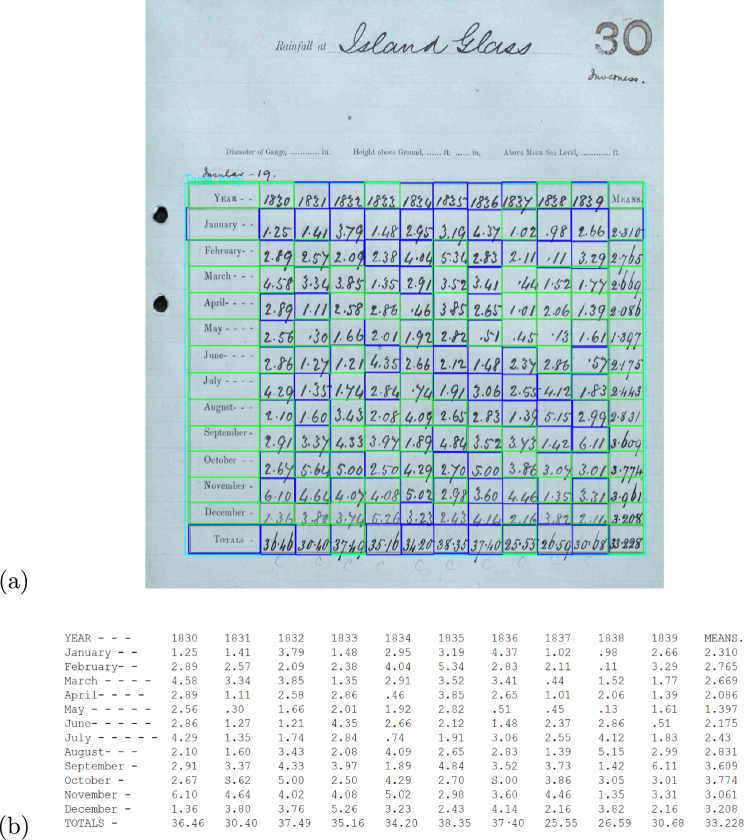
Visualization of the tabular data reconstruction process using a *UK* logbook image. (a) Table Structure Recognition output: The cells highlighted in blue represent the predictions made by CascadeTabnet, while those in green denote the newly generated cells derived from the coordinates of the blue-highlighted cells. (b) Tabular data reconstruction output: Content extraction using TrOCR-ctx and coordinate-based alignment for final table reconstruction. The performance of the TSR model on this image is 0.997 wF1-score, while the text extraction performances are 0.969 and 0.874 F1-scores at character and token levels, respectively

**Fig. 6:**
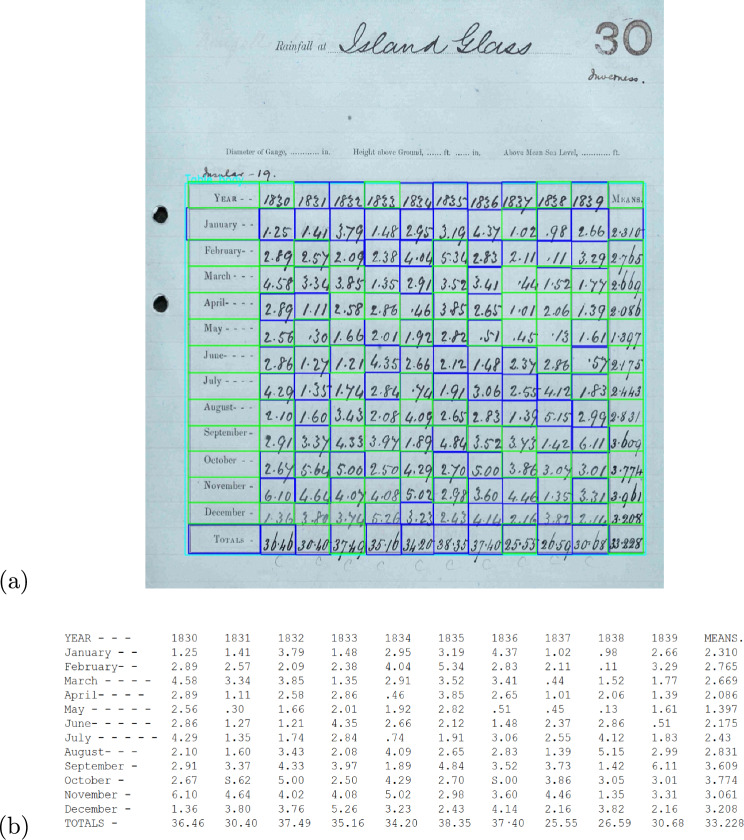
Visualization of the tabular data reconstruction process using a *Ben Nevis, UK* logbook image. **(a)** Table Structure Recognition output: The cells highlighted in blue represent the predictions made by CascadeTabnet, while those in green denote the newly generated cells derived from the coordinates of the blue-highlighted cells. **(b)** Tabular data reconstruction output: Content extraction using TrOCR-ctx and coordinate-based alignment for final table reconstruction. The performance of the TSR model on this image is 0.989 wF1-score, while the text extraction performances are 0.974 and 0.927 F1-scores at character and token levels, respectively

## Data Availability

Dataset and source code are released publicly via GitHub (code) and Zenodo (data) using an open source licence (BSD for source code, creative commons for data). The URI to these sites can be found within the paper.

## Appendix

See Figures 5 and 6.

## Annotation guidelines

The guidelines for crowd annotators in this task were designed to provide clear instructions for working with table images containing highlighted cells. The primary objective for annotators is to accurately correct cell boundaries and transcribe the highlighted content. For consistency and precision in annotating various cell types, detailed instructions, as outlined in Table 7, are provided. The task encompasses several possible scenarios, each with specific actions to guide annotators in handling different cell boundaries and content configurations.

1. When the highlighted cell boundaries accurately enclose the text (i.e., all text is contained within the cell boundary lines), transcribe the text directly within the highlighted cell.
2. When the highlighted cell boundaries are inaccurate (e.g., the text extends beyond the boundary lines), adjust the cell boundaries to fully contain the text and then transcribe the content.
3. If a highlighted area merges multiple cells into a single box, remove the highlighted box, create individual boxes for each separate cell, adjust boundaries as necessary, and transcribe the content within each cell.

**Table 7:** Guidelines on the type of cells to transcribe

Type of cells Cells	Examples	Transcription
If the text in the cell is **easy to read and transcribe**, simply transcribe the content as it appears.		1923
If the highlighted cell **covers multiple distinct text regions**, adjust the cell boundary by adding new cells according to the table structure and transcribe each distinct text region within its own cell.		Cell 1: 1, Cell 2: 9.16
If the highlighted cell **only partially covers a text region**, correct the cell boundaries in line with the table structure, then transcribe the text within each corrected cell.		229.18
If a single word or group of words spans across multiple highlighted cells, **combine these cells** by adjusting boundaries so that each cell contains a single, complete text region. Then, transcribe the text accordingly.		Information
If any part of the highlighted text **cannot be easily transcribed**, transcribe the cell as ‘Å‘. This will alert an expert to review the cell later for clarification.		Å

**Table 8:** Parameters for training the CascadeTabNet model

Description	Value
Input image size (height x width)	1024x1024
Backbone model used for feature extraction	ResNet-50
Number of output channels in the last layer of the backbone	256
Number of input channels	256
Number of fully connected (FC) layers	2
Number of output channels for each FC layer	1024
Number of stages in the cascade	3
Region Proposal Network (RPN) output threshold	2000
RPN minimum positive IoU threshold	0.3
Number of object classes (table or background)	2
Learning rate of the optimizer	0.005
Loss function for classification	Cross Entropy
Loss function for bounding box regression	Smooth L1

**Table 9:** Parameters for training the TrOCR model

Hyperparameter	Value
Image Size	(120, 80)
Maximum Text Length	190
Optimizer	Adam
Loss Function	CrossEntropyLoss

## Footnotes

1 https://github.com/gyanendrol9/context-aware_table_extraction, https://zenodo.org/records/15730546.

2 https://deepmind.google/models/gemini/flash/.

3 https://openai.com/index/hello-gpt-4o/.

4 https://digital.nmla.metoffice.gov.uk.

5 https://libguides.library.noaa.gov.

6 https://catalog.archives.gov.

7 http://archives-climat.fr.

8 https://www.appen.com.
